# Virulence factors released by extracellular vesicles from *Cryptococcus neoformans*

**DOI:** 10.3389/fcimb.2025.1572520

**Published:** 2025-05-14

**Authors:** Wenhao Xiao, Huiqiang Lu, Bowei Jiang, Yaqi Zheng, Puwen Chen, Xiaotong Liu, Junyun Huang

**Affiliations:** The First Clinical Medical College of Gannan Medical University, Ganzhou, China

**Keywords:** *Cryptococcus neoformans*, extracellular vesicles, virulence factors, mechanism, therapeutic potential

## Abstract

*Cryptococcus neoformans*, a prominent opportunistic pathogen, is equipped with unique mechanisms to evade host immune defenses, notably through its capsule and the secretion of extracellular vesicles (EVs). Despite significant understanding of its pathogenesis, the precise role of EVs in virulence and their molecular components remain underexplored. This review synthesizes current research on the virulence factors encapsulated within EVs of *Cryptococcus*, highlighting their contribution to fungal survival and pathogenicity. By analyzing the biochemical composition of these vesicles, we found the presence of enzymes (e.g., Urease, laccase), toxins (e.g. Melanin), and genes (e.g. Ssa1) associated with pathogenicity factors. Furthermore, we discuss the implications of these findings for developing therapeutic interventions. This work advances the field by providing a comprehensive overview of EV-mediated mechanisms in *Cryptococcus*, offering new insights into potential targets for antifungal strategies.

## 1 Introduction

*Cryptococcus neoformans*, as a popular subject of fungal research in recent years, holds extremely important research value (Duarte and Rodrigues, 2024). The cryptococcal meningitis it caused is particularly life-threatening for many immunocompromised HIV-infected individuals and is also a burden on healthcare systems worldwide (Rajasingham et al., 2017). Extracellular vesicles, as a heterogeneous cell-derived membrane structure, are involved in the exchange of proteins, lipids, and genetic material between cells (van Niel et al., 2018); in *Cryptococcus neoformans*, these vesicles have been found to carry and transmit various virulence-related molecules, such as enzymes, toxins, and immune regulatory factors. These factors have a destructive effect on host cells, promoting the colonization, invasion, and dissemination of the pathogen. Recently, Rizzo et al. utilized cryo-electron microscopy (cryo-EM) and cryo-electron tomography (cryo-ET) to analyze the structure of native extracellular vesicles (EVs) in *Cryptococcus* spp. Their findings revealed a novel structural model for *Cryptococcus*, wherein the outer layer comprises glucuronoxylomannan (GXM), and the inner lipid bilayer is densely populated with proteins and encased within a mannoprotein-rich fibrous matrix (Rizzo et al., 2021). Baker et al. (2024) discovered that the melanin in *Cryptococcus neoformans* can chelate calcium ions, limiting the availability of calcium for the bivalent bridges between polysaccharide subunits required for capsule formation.Under chelation, darkened cells shed a large amount of polysaccharides and reduce their ability to integrate secreted polysaccharides into the growing capsule. This change may have profound negative impacts on the host’s immune response (Baker et al., 2024). Chadwick et al. (2024) also discovered through genomic sequencing and other methods that wild strains of *Cryptococcus neoformans* adapt to the CO2 levels within the host based on the combined effects of multiple genetic loci. They proposed new insights into how current antifungal drugs induce the evolution of *Cryptococcus neoformans*, suggesting that future drugs could be developed with consideration of the CO2 levels within the host as a guide for enhancing the tolerance of *Cryptococcus neoformans* (Chadwick et al., 2024). A deep understanding of the release mechanisms of extracellular vesicles in *Cryptococcus neoformans* and the virulence factors they carry is of great significance for developing new therapeutic strategies against cryptococcosis.

## 2 Biogenesis of extracellular vesicles and immune response after macrophage phagocytosis

### 2.1 Biogenesis and types of extracellular vesicles

**Figure 1:**
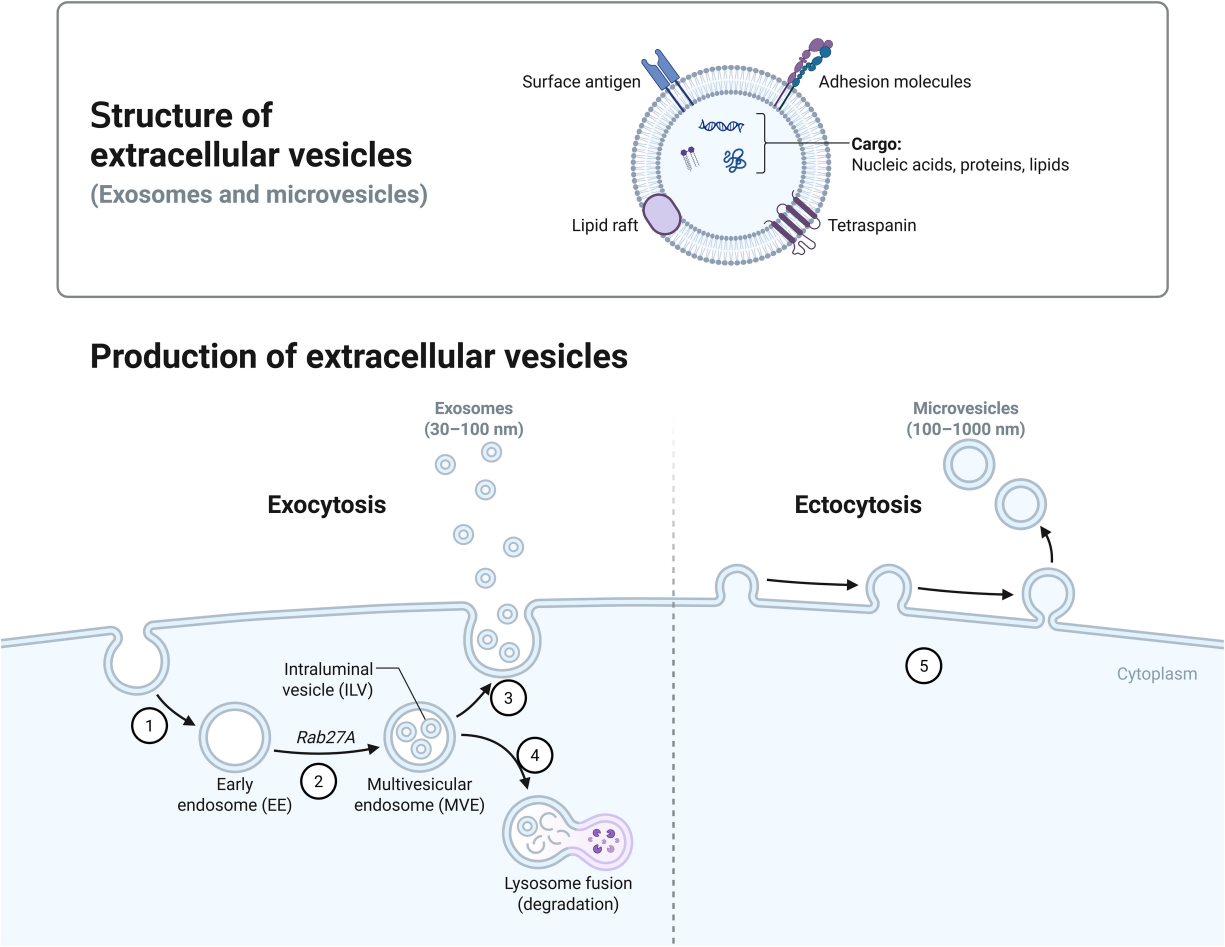
Production of extracellular vesicles. Bounded by the dotted line, **Figure 1** shows two different biogenesis pathways for extracellular vesicles. On the left (1) Early endosomes are formed by endocytosis. (2) Rab27a is involved in the docking of plasma membranes with MVBs. (3) Exosomes are released after the MVB fuses with the plasma membrane. (4) Lysosome is involved in the degradation of goods within the MVB. On the right: (5) Microvesicles are formed by budding directly through the cell membrane.

Extracellular vesicles (EVs) are small membrane-bound particles released by cells into the extracellular environment.These vesicles play a crucial role in intercellular communication, transporting proteins, lipids, and nucleic acids. Extracellular vesicles secreted by mammals can be broadly classified into several categories, including exosomes, microvesicles, and apoptotic bodies, each differing in size, biogenesis, and function (See **Figure 1**) (Piffer et al., 2021; Reis et al., 2021). (1) Exosomes: Exosomes typically have a diameter ranging from 30 to 150 nanometers, and their formation process involves the endosome formed after endocytosis.The small vesicular structures formed inside the endosome are called multivesicular bodies (MVBs). The vesicles contained within these MVBs are released into the extracellular space by fusing with the cell membrane, becoming exosomes.This process involves various proteins, such as the ESCRT complex (Endosomal Sorting Complex Required for Transport), Rab27a[(Plays a role in the docking of plasma membranes with MVBs)],TSG101, and Alix, which help in the scission of vesicles and the selective packaging of certain proteins, RNA, and other molecules.(2) Microvesicles: The diameter of microvesicles is typically between 100–1000 nanometers, and they are formed directly through the outward budding and fission of the cell membrane. During this process, the distribution of phospholipids in the cell membrane changes, for example, phosphatidylserine (PS) flips from the inner layer to the outer layer of the cell membrane. This is regulated by phosphatidylserine flippases and phosphatidylserine scramblases.Moreover, the reorganization of the cytoskeleton also plays a crucial role in the formation of microbodies.(3) Apoptotic bodies: Apoptotic bodies are formed during the process of apoptosis and are usually larger than 1000 nanometers. They contain cell debris, such as organelles, DNA fragments, etc. During apoptosis, the nucleus and organelles of the cell break apart, the cell membrane invaginates to form bubble-like structures, and ultimately, the cell body ruptures to form apoptotic bodies (Ostrowski et al., 2010; Maas et al., 2017; Gurunathan et al., 2019; Popa and Stewart, 2021). Compared to mammals, Fungal cells can produce extracellular vesicles (EVs) through two distinct biogenesis pathways: endosomal-derived exosomes and plasma membrane-derived ectosomes. Exosomes originate from the endocytic pathway, while ectosomes are generated through direct budding of the plasma membrane (Rodrigues et al., 2015; Nenciarini and Cavalieri, 2023).

### 2.2 Immune response after macrophage phagocytosis

**Figure 2:**
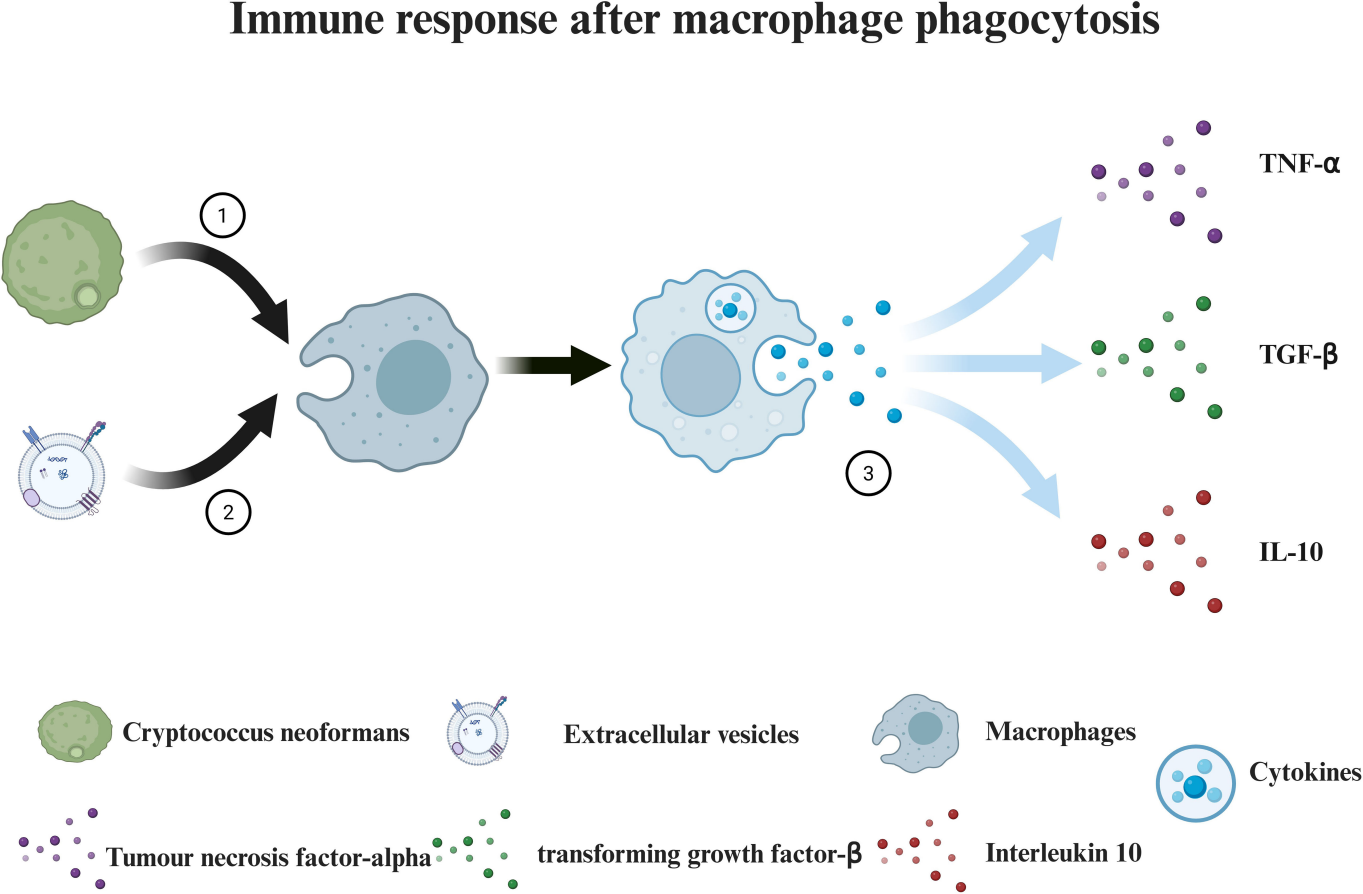
Immune response after macrophage phagocytosis. (1) Macrophages engulf *Cryptococcus neoformans*. (2) Macrophages engulf extracellular vesicles. (3) Macrophages release various cytokines (e.g., TNF-α, TGF-β, and IL-10).

Macrophages are pivotal components of the immune system, distinguished by their capacity to phagocytose pathogens and eliminate cellular debris. During Cryptococcus infection, macrophages rapidly recognize and internalize pathogen-secreted extracellular vesicles (EVs) via endocytosis, a mechanism that systemically regulates phagocyte functionality (Doherty and McMahon, 2009; Oliveira et al., 2010). Oliveira et al. demonstrated that *Cryptococcus neoformans*-derived EVs activate murine RAW 264.7 macrophages in a dose-dependent manner, markedly stimulating nitric oxide (NO) synthesis, upregulating cytokine secretion [including TNF-α(Tumour necrosis factor-alpha), TGF-β(transforming growth factor-β), and IL-10(Interleukin 10)], and enhancing both phagocytic and bactericidal activities (See **Figure 2**). Complementarily (Oliveira et al., 2010), Zhang et al. identified that EVs secreted by bone marrow-derived macrophages (BMDMs) following *C. neoformans* engulfment—termed BM-EVs—trigger immune-associated signaling pathways in naïve BMDMs, inducing polarization toward the M1 phenotype and ultimately reducing fungal burden in murine infection models (Zhang et al., 2021).

The release of extracellular vesicles is not only a way for cells to eliminate excess or harmful substances but also an important means of intercellular communication. Through these vesicles, cells can transmit biomolecules such as proteins, lipids, RNA, and DNA, influencing the function and behavior of distant or neighboring cells. Therefore, EVs are receiving increasing attention in biomedical research, particularly for their potential applications in disease diagnosis, treatment, and regenerative medicine.

## 3 Extracellular vesicle-secreted virulence factors of *Cryptococcus neoformans*

**Figure 3:**
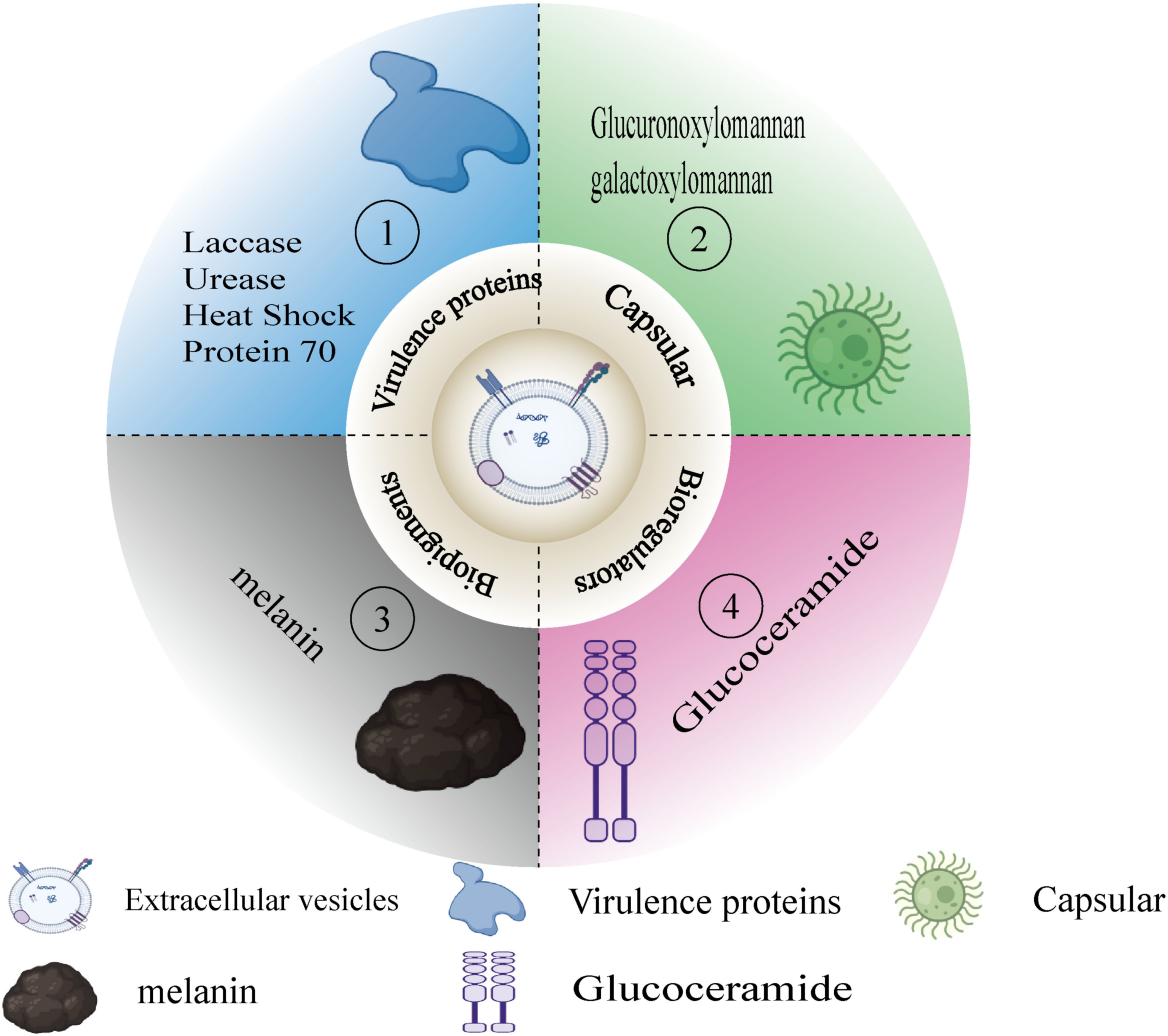
Virulent substances in EVs. **Figure 3** classifies the virulent substances in the EVs that appear in this paper. (1) Virulence proteins(e.g.,laccase, urease). (2) Capsule (e.g.,GXM,GalXM). (3) Biopigments (e.g.,melanin). (4) Bioregulators (e.g., glucoceramide).

According to sources, the definition of virulence factors is indeed quite complex and lacks uniformity. Nevertheless, there is a general consensus that virulence factors can be seen as substances or elements within pathogens that may cause damage to the host (Pirofski and Casadevall, 2015). These factors are not only the weapons with which pathogens attack the host but also key tools for their survival and transmission. The secretion of virulence factors, particularly the attack on specific host cell sites, demonstrates the complex interaction mechanisms between pathogens and hosts (Casadevall and Pirofski, 2003). In specific pathogens, such as *Cryptococcus neoformans*, the types of virulence factors can be roughly divided into four categories based on their functions and mechanisms of action. First are virulence proteins, such as urease and phospholipase B, which promote pathogen invasion and survival by disrupting the structure and function of host cells. Second are capsule polysaccharides, like GXM and GalGXM, which enhance the pathogen’s resistance by inhibiting the host’s immune response. The third category is other biological regulatory factors, such as glucosylceramide, which interfere with normal cellular functions by regulating the host’s cell signaling processes. Finally, there are biological pigments, such as melanin, which not only protect pathogens from the host’s immune system but may also directly participate in the destruction of cellular structures (See **Figure 3**). When we narrow our research focus to virulence factors secreted by extracellular vesicles, the number of identifiable virulence substances decreases. These vesicles are tiny packages released by pathogens into the host, containing various molecules that can interfere with or disrupt host cell functions, and Extracellular vesicles (EVs) exhibit increased hydrodynamic diameter, higher concentrations of virulence factors, and enhanced immunomodulatory activity in host organisms under nutrient-limited conditions (Marina et al., 2020). Although the number of known virulence factors of this type is currently limited, their role is extremely critical because this secretion mechanism allows pathogens to regulate host cell behavior from a distance without direct contact. In summary, the study of virulence factors not only helps us gain a deeper understanding of the interactions between pathogens and hosts but also offers possibilities for developing new therapeutic methods. By intervening and blocking specific virulence factors, we can effectively weaken the pathogenicity of pathogens, providing new strategies for treating various diseases caused by pathogens.

### 3.1 Virulence proteins

#### 3.1.1 Laccase

Laccase, as a multicopper oxidase, is widely present in various organisms such as higher plants, insects, fungi, and bacteria (Giardina et al., 2010). In *Cryptococcus neoformans*, laccase is not only an important virulence factor related to the cell wall but also participates in various biochemical processes, including the catalysis of L-3,4-dihydroxyphenylalanine (l-DOPA), thereby contributing to melanin synthesis.In addition, laccase also has multiple biological functions, including protecting host cells from harsh environments, toxic reactive oxygen and nitrogen species, phagocytic killing by macrophages, immune system attacks, and antifungal compounds (Nosanchuk and Casadevall, 2003; Eisenman et al., 2007; Coman et al., 2013). Rodrigues et al. (2008) conducted a detailed characterization of laccase activity in *Cryptococcus neoformans* extracellular vesicles through proteomics and biochemical assays. Research has found that these vesicles can effectively deliver concentrated *Cryptococcus* protein virulence factors to the effector cells and tissues of the host, thereby exerting their pathogenicity (Rodrigues et al., 2008). Furthermore, Panepinto et al. (2009) observed a complete absence of vesicles and the cessation of cell wall laccase secretion by knocking down the RNAi expression of the protein complex SEC6 involved in the fusion of extracellular vesicles with membrane polarization. This change led to a decrease in urease and extracellular polysaccharide levels in mice, as well as a significant increase in mouse survival rates (Panepinto et al., 2009). In the *in vivo* environment, laccase exhibits a melanin-independent anti-macrophage phagocytosis effect. Laccases with ferroxidase activity can reduce the formation of hydroxyl radicals in macrophages and dominate the non-soluble exocytosis of macrophages without the addition of melanin substrates (Liu et al., 1999; Frazão et al., 2020). A study by Sabiiti et al. (2014) further validated this with 65 clinical isolates, finding that *Cryptococcus* strains with higher laccase activity showed higher macrophage phagocytic uptake rates and lower *Cryptococcus* clearance rates (Sabiiti et al., 2014). These research findings not only enhance our understanding of the function of laccase in *Cryptococcus neoformans* but also provide a potential biological basis for developing new therapeutic strategies against this pathogen. By targeting laccase and its associated extracellular vesicle transport mechanisms, new antifungal treatment methods may be developed, thereby effectively controlling or eliminating infections caused by this pathogen.

#### 3.1.2 HSP70

Heat shock proteins (Hsp) are a group of key stress-induced proteins that are widely involved in protein folding, maturation, and homeostasis maintenance (Kampinga et al., 2009). In various organisms, Hsp plays a crucial role in cellular physiology and stress response by promoting proper protein folding, degradation processes, and preventing protein aggregation (Chan and Groisman, 2024). In 2011, Antonio first reported Hsp70 protein as a component of fungal extracellular vesicles (EVs) (De Maio, 2011). Subsequently, in 2021, Fabio and others further confirmed the critical role of Hsp70 in the formation of all fungal EVs through shotgun proteomics analysis (Parreira et al., 2021). In *Cryptococcus neoformans*, cytoplasmic Hsp70 protein directly interacts with DNA within the 5′-UAS region of the laccase gene to jointly activate gene expression, promoting melanin production in the serotype D strain JEC21. However, a study by Eastman et al. (2015) indicated that in the serotype A strain H99, cytoplasmic Hsp70 protein does not regulate the expression of laccase associated with melanin synthesis. Instead, it affects primary defense against *Cryptococcus* infection by promoting non-protective early M2 activation of pulmonary macrophages, interfering with the innate immune response, and maintaining the normal development of adaptive immunity (Zhang et al., 2006; Eastman et al., 2015). Moreover, mutants lacking the homologous gene of Hsp70 (Ssa1) showed reduced virulence in mouse infection models, indicating that the cytoplasmic Hsp70 protein produced by Ssa1 on the fungal surface may be involved in inhibiting the production of nitric oxide (NO) in macrophages, which is a key factor in the innate immunity of pulmonary epithelial cells (Zhang et al., 2006; Silveira et al., 2013). Hsp70 not only plays an important role in the survival and pathogenic mechanisms of pathogens but also serves as a novel immunogenic protein, making it a potential diagnostic marker or vaccine target for cell and humoral responses in mice with pulmonary cryptococcosis and cryptococcal meningitis (Kakeya et al., 1997, 1999; Steen et al., 2003; Firacative et al., 2018). Based on the role of Hsp70 in the virulence of *Cryptococcus* pathogens, Elhassan et al. (2021) employed epitope-based immunoinformatics methods to analyze homologous proteins of highly affine T cells and B cells within the Hsp70 family proteins in the Immune Epitope Database (IEDB), thereby designing a vaccine targeting highly conserved *Cryptococcus neoformans* peptides (Elhassan et al., 2021). These studies not only reveal the diverse roles of Hsp70 in fungal biology but also provide important scientific evidence for the development of new antifungal therapies.

#### 3.1.3 Urease

Urease, as a nutritional enzyme, is widely present in various bacterial pathogens and acts as a general virulence factor. This enzyme hydrolyzes urea to produce ammonia, which is crucial for various organisms, thereby playing a key role in the nitrogen cycle within the organism. During the process of *Cryptococcus neoformans* infection, the role of urease is particularly significant; it not only interferes with the acidification of phagosomes but also promotes the pathogen’s invasion into the host’s brain (Shi et al., 2010; Fu et al., 2018). Studies have shown that after infecting the host, *Cryptococcus neoformans* produces a large amount of urease, a characteristic that has been used in the diagnosis of cryptococcosis (Zimmer and Roberts, 1979). In-depth research has revealed the close connection between urease and another key virulence factor in *Cryptococcus neoformans*—melanin (Baker and Casadevall, 2023). Urease is typically released into extracellular vesicles, where it breaks down urea to produce ammonia, which then acts distally as a gas through diffusion. Furthermore, the ammonia produced by urease activity increases the pH of the phagolysosome, thereby promoting melanin formation. This pH-dependent mechanism not only delays the yeast form replication of *Cryptococcus neoformans* but also reduces damage to host macrophages and extends the pathogen’s residence time within the cells. Further research indicates that enhanced melanization supports the persistent presence of *Cryptococcus* cells within phagosomes and promotes widespread dissemination in the brain through a Trojan horse-like strategy. Moreover, the accumulation of melanin in the cell wall may act as a feedback mechanism, inhibiting urease production associated with vesicles by reducing the release of urease in extracellular vesicles (Rodrigues et al., 2008; Shi et al., 2010; Baker and Casadevall, 2023). Urease also promotes non-lytic exocytosis by influencing the adaptability of phagosomes in mammalian models, while delaying intracellular replication and reducing damage to the phagolysosomal membrane, thereby facilitating the spread of *Cryptococcus* during its transport within macrophages (Fu et al., 2018). When urease needs to be released into the extracellular space in secretory vesicles, its release dosage depends on the activity of the extracellular vesicles. Therefore, it can be inferred that extracellular vesicles act as “transport bags” for urease, playing a key role in the transmission and release of urease virulence (Yoneda and Doering, 2006; Rodrigues et al., 2008). It is evident that the urease released by extracellular vesicles plays a decisive role in mediating the pathogenicity of *Cryptococcus neoformans*. These findings not only enhance our understanding of the pathogenic mechanisms of *Cryptococcus neoformans* but also provide new perspectives for therapeutic strategies targeting such pathogens.

### 3.2 Capsule polysaccharides

#### 3.2.1 GXM

Glucuronoxylomannan (GXM) is a key component of the *Cryptococcus neoformans* polysaccharide capsule, and it exerts regulatory effects on the host immune system through various mechanisms. GXM exhibits its immunomodulatory function by inhibiting the secretion of pro-inflammatory cytokines by human monocytes and restricting leukocyte migration. Additionally, GXM achieves immune evasion by resisting macrophage phagocytosis, further enhancing its survival ability within the host (Kozel et al., 1988; Ellerbroek et al., 2004). Extracellular vesicles, as an important immune-stimulating component of *Cryptococcus neoformans*, have the ability to activate macrophage inflammatory responses. This activation is mainly manifested in promoting the production of tumor necrosis factor α (TNF-α) and interleukin 10 (IL-10), thereby enhancing the antifungal activity of macrophages (Oliveira et al., 2010). The main function of extracellular vesicles is to facilitate the exchange of proteins, lipids, and genetic material between cells. The study by Rodrigues et al. (2007) found that extracellular vesicles produced by *Cryptococcus neoformans* contain immunologically active lipids, which can encapsulate GXM and release it into the extracellular environment through the cell wall (Rodrigues et al., 2007). When macrophages ingest *Cryptococcus neoformans*, the phagosome membrane may be damaged, leading to a disruption of cytoplasmic continuity and the accumulation of polysaccharide vesicles, further causing functional impairment of macrophages and a decline in host immune function (Tucker and Casadevall, 2002). Moreover, the accumulation of GXM from different serotypes may inhibit the production of the protective response factor nitric oxide (NO) induced by vesicles during *Cryptococcus neoformans* infection (Rossi et al., 1999; Oliveira et al., 2010). Oliveira et al. (2010) observed that the differences in the composition of vesicles secreted by *Cryptococcus neoformans* affect the ability of macrophages to produce cytokines and their corresponding stimulatory responses (Oliveira et al., 2010). Further research indicates that when macrophages phagocytose extracellular vesicles containing GXM, GXM can inhibit the response of CD4+ T lymphocytes through an IL-10-dependent mechanism, thereby promoting the growth of yeast cells *in vitro* (Mariano Andrade et al., 2003). Yauch et al. (2006) also found that whether activated through antigen-presenting cells (APC) with antigen or directly by mitogen, GXM can directly inhibit T lymphocyte proliferation and reduce cellular immune responses in mice and humans after T cells are absorbed by dendritic cells (DC) (Yauch et al., 2006). The findings of the aforementioned study have provided us with new insights into the role of GXM in host immune regulation and have also offered potential targets for the development of new therapies against cryptococcal infections.

#### 3.2.2 GalXM

GalXM, as a minor component (approximately 10%) of *Cryptococcus neoformans* capsular polysaccharide, has long been overlooked. However, with the in-depth study of GXM pathogenicity and the immune mechanisms of GalXM in the body, GalXM has been found to potentially be a more effective immunomodulatory factor than GXM (Pericolini et al., 2006; De Jesus et al., 2007). The study by Pericolini et al. (2006) demonstrated that GalXM can induce T cell apoptosis by recruiting and activating death receptors and the key initiator caspase-8 in the extrinsic apoptotic pathway, leading to DNA fragmentation and the upregulation of surface molecules Fas/FasL that are involved in inducing apoptosis. This mechanism leads to immune suppression in the human body, indicating that GalXM plays a crucial role in regulating the host immune response (Pericolini et al., 2006). The study by Villena et al. (2008) further found that GalXM not only induces the primary production of tumor necrosis factor TNF-α but also induces higher levels of nitric oxide synthase (iNOS) and other inflammatory factors such as NO in RAW macrophages. These findings confirm that GalXM, compared to GXM, requires a lower dose to upregulate Fas expression on the surface of macrophages and induce their apoptosis, and it is more effective in enhancing the survival rate of *Cryptococcus neoformans* within macrophages (Villena et al., 2008). De Jesus et al. (2009) found the components of GalXM in the isolated extracellular vesicle components during their study and observed the accumulation of GalXM in budding *Cryptococcus neoformans* cells using immunofluorescence labeling techniques. These findings support the hypothesis that GalXM may mediate the colonization and invasion of *Cryptococcus neoformans* through extracellular vesicles. Extracellular vesicles, as an important molecular transport mechanism, may play a crucial role in the pathogenicity of *Cryptococcus neoformans* (Rodrigues et al., 2007; De Jesus et al., 2009). In summary, the aforementioned studies not only reveal the complexity of GalXM in the interaction between *Cryptococcus neoformans* and its host but also provide an important scientific basis for the development of novel therapeutic strategies.

### 3.3 Biological regulatory factors

#### 3.3.1 GlcCer

Glucosylceramide (GlcCer) is an antigenic sphingolipid found on the surface of fungal cells and can induce an antibody response in patients with cryptococcosis or in mice. In 2000, Rodrigues et al. successfully localized GlcCer in *Cryptococcus neoformans* for the first time, finding that it is primarily distributed in the cell wall and plasma membrane, and is enriched at the budding sites of secretory cells (Rodrigues et al., 2000). Rittershaus et al. (2006) used recombinant PCR technology to knock down the gene encoding GlcCer synthase (GCS), revealing a direct link between the unsaturation of the sphingosine backbone in GlcCer and the ability of *Cryptococcus neoformans* to establish virulence (Rittershaus et al., 2006). Subsequently, Rodrigues et al. (2007) conducted high-performance thin-layer chromatography (HPTLC) lipid analysis on the vesicle lower phase after high-speed centrifugation, further confirming the enrichment of GlcCer in vesicle lipid extracts (Rodrigues et al., 2007). Based on this, Raj et al. (2017) used genetic engineering techniques to alter the chemical structure of GlcCer in *Cryptococcus neoformans* and performed biophysical characterization of the purified GlcCer vesicles.The study found that the unsaturation at carbon position 8 (C8) and the methylation at carbon position 9 (C9) of the GlcCer sphingosine backbone significantly increased the sensitivity of *Cryptococcus neoformans* to membrane stressors, resulting in increased membrane permeability and thereby inhibiting the growth of the pathogen within host macrophages (Raj et al., 2017). Mor et al. (2016), on the other hand, took a different approach by purifying GlcCer from the non-pathogenic fungus Candida utilis and injecting it into the peritoneal cavity of mice infected with *Cryptococcus neoformans*. The experiment found that the purified GlcCer could effectively enhance the mice’s resistance to lethal nasal invasion by *Cryptococcus neoformans*, hindering the spread of the fungus to the brain. This finding indicates that GlcCer not only plays a crucial role in the virulence formation of *Cryptococcus* neoformans but also shows its potential as a vaccine against cryptococcosis (Mor et al., 2016). Whether it is the pathogenic mechanism of GlcCer-containing vesicles or the study of vaccines, it not only deepens our understanding of GlcCer’s role in the pathogenic mechanism of *Cryptococcus neoformans* but also highlights the potential value of GlcCer as a therapeutic target for *Cryptococcus neoformans*. This provides important scientific evidence for the development of new antifungal vaccines and therapeutic strategies.

### 3.4 Biological pigments

#### 3.4.1 Melanin

Melanin is a brown or black hydrophobic high molecular weight pigment with a negative charge, known to play a protective role in various organisms. As early as 1995, Wang and colleagues’ research first revealed that melanin can protect *Cryptococcus neoformans* yeast cells from phagocytosis by macrophages (Wang et al., 1995). Subsequently, further studies by Casadevall and others found that melaninization in *Cryptococcus neoformans* not only enhances its resistance to macrophages but also protects the cells from UV radiation, oxidative stress, and extreme temperatures (Rosas and Casadevall, 1997; García-Rivera and Casadevall, 2001). The presence of laccase promotes the rapid conversion of the L-3,4-dihydroxyphenylalanine (L-DOPA) substrate into melanin. Eisenman (2009), in studying the mechanism of melanin synthesis, observed vesicles similar in size to melanin particles depositing at the bottom of the grid by co-incubating L-DOPA with purified extracellular vesicles and applying quasi-elastic light scattering (QELS).Based on these observations, Eisenman proposed a hypothesis that the melanin synthesis in *Cryptococcus neoformans* might primarily occur in extracellular vesicles (Eisenman et al., 2009). Recent studies, such as the work by De Sousa et al. (2022), have conducted dynamic light scattering (DLS) analysis on extracellular vesicles from clinical isolates of 65 *Cryptococcus neoformans* strains, finding a significant correlation between the ergosterol content in the vesicles and visual melanization scores.In addition, they also found that the number of vesicles is related to faster melanization and greater capsule thickness in rich media (de Sousa et al., 2022). Baker’s (2023) study further demonstrates the mutual regulatory mechanisms between melanin and other virulence factors such as urease. Urease increases pH by producing volatile ammonia, which affects neighboring cells from a distance, thereby promoting melanization. This interaction, in turn, reduces urease activity by inhibiting the secretion of vesicles carrying the enzyme, resulting in fungal cells with different characteristics and adaptations, thereby promoting the survival and spread of cryptococcosis at various stages (Baker and Casadevall, 2023). Overall, these studies reveal the complex role of melanin in the pathogenic mechanisms of *Cryptococcus neoformans* and highlight the importance of its synthesis in extracellular vesicles for enhancing pathogen virulence and survival rates within the host. (**Table 1** summarizes the above).

**Table 1:** Summary of extracellular vesicle virulence factors.

Numbering	Virulence factors	Function	References
1	Laccase	1. Catalyzes the synthesis of melanin 2. Affect phagocytosis rate	(Liu et al., 1999; Eisenman et al., 2007; Frazão et al., 2020)
2	HSP70	1. Promote macrophage M2 activation 2. Inhibit NO production	(Silveira et al., 2013; Eastman et al., 2015)
3	Urease	1.Reduce lysosomal membrane damage 2.Helps invade brain parenchyma	(Shi et al., 2010; Fu et al., 2018)
4	GXM	1. Inhibit cytokine production 2. Damaged macrophages	(Tucker and Casadevall, 2002; Oliveira et al., 2010)
5	GalXM	1. Inducing T cell apoptosis 2. Induction of TNF-α production	(Pericolini et al., 2006; Villena et al., 2008)
6	GlcCer	1. Promote the development and growth of mycelium 2. Increase the permeability of the cell membrane	(Rittershaus et al., 2006; Raj et al., 2017)
7	Melanin	1. Prevent the oxidative burst of phagocytes	(Wang et al., 1995)

## 4 The potential therapeutic applications of virulence factors secreted by extracellular vesicles of *Cryptococcus neoformans*

Due to the overuse of antimicrobial drugs, an increasing number of pathogenic bacteria have developed resistance to one or more antimicrobial agents.This phenomenon poses a severe challenge to traditional antimicrobial treatment strategies, indicating an urgent need to develop new antifungal therapies. In the search for new treatment methods, one strategy is to target the virulence factors of pathogens, rather than merely killing or inhibiting the microorganisms themselves. This approach has the potential advantages of expanding the microbial target library, protecting the host’s endogenous microbiome, and exerting less selective pressure, which may reduce the development of resistance (Clatworthy et al., 2007). Fungal extracellular vesicles (EVs) are complex structures that contain various components with pathogenic and immunogenic properties, capable of triggering the host’s immune response. Research on *Cryptococcus neoformans* indicates that the virulence factors carried and secreted by its extracellular vesicles, such as capsule polysaccharide (GXM), extracellular enzymes, and melanin, are potential targets for antifungal therapy (Gil-Bona et al., 2015).

In *Cryptococcus neoformans*, the biosynthesis of the capsule polysaccharide GXM involves multiple enzymes and transport proteins. We found that it is closely related to two enzymes: one is GDP (guanosine diphosphate), which is coupled with mannose through glycan biosynthesis reactions and has two transport proteins, Gmt1 and Gmt2, that facilitate the *in vitro* transport of GDP-mannose. Strains lacking both Gmt1 and Gmt2 exhibit impaired capsule biosynthesis, protein glycosylation processes, and reduced virulence. In humans and other mammalian hosts, no form of GDP-mannose transport protein is expressed, highlighting its potential as a target for antimicrobial therapy (Wang et al., 2014). Another one is Cmt1 and its associated enzymes. The lack of Cmt1 in mammalian and other animal hosts eliminates its enzymatic activity, but does not prevent capsule formation or loss of virulence. This suggests the presence of other compensatory factors involved in the biosynthesis of GXM. Although data have not yet confirmed its functional role in *Cryptococcus neoformans*, it does not preclude its potential as a target for future *Cryptococcus* treatment (Sommer et al., 2003).

In addition, melanin is also an attractive therapeutic target. In animal model experiments, monoclonal antibodies targeting melanin were able to extend the survival period of infected mice and reduce fungal loads in different organs. This suggests that the passive immunization strategy using melanin monoclonal antibodies may have therapeutic potential (Rosas et al., 2001). Recent studies, such as those by Figueiredo et al. (2021), have shown that the developed monoclonal antibody Mab targeting the cell wall chitin oligomer not only increases the sensitivity of *Cryptococcus neoformans* to the primary antifungal drug amphotericin B but may also alter melanization by inducing the disintegration of the cell wall matrix through binding to the surface of the fungus (Nosanchuk et al., 2015; Figueiredo et al., 2021).

Emerging evidence highlights the significant therapeutic potential and scientific relevance of *Cryptococcus* extracellular vesicles (EVs). Colombo et al. demonstrated that enrichment of glucuronoxylomannan (GXM)-containing EVs significantly delayed mortality rates in *Cryptococcus neoformans*-infected Galleria mellonella models (Colombo et al., 2019). Furthermore, Freitas et al. systematically examined the immunomodulatory properties of *Cryptococcus* EVs for therapeutic applications and assessed their viability as novel vaccine candidates (Freitas et al., 2019).

## 5 Conclusion

The field of *Cryptococcus neoformans* treatment has undergone a significant paradigm shift in response to escalating fungal drug resistance. Contemporary research has moved beyond traditional single-factor approaches to systematically investigate: (1) global metabolic network regulation, and (2) cooperative resistance mechanisms between virulence factors. This integrated approach yields dual benefits: it establishes causal relationships between metabolic adaptation and virulence regulation, informing novel therapeutic strategies, while also enabling rational design of combination therapies for resistant infections. Notably, the conserved stress-response pathways discovered in *C. neoformans* provide translational insights for managing other clinically important fungi, including Aspergillus and Candida species. These developments promise to enhance both cryptococcosis treatment and broader antifungal drug development.
